# Low-dose metformin and PEN2-dependent lysosomal AMPK activation: benefits outnumber side effects

**DOI:** 10.1038/s41392-022-01040-9

**Published:** 2022-06-04

**Authors:** Longlong Liu, Pradeep Kumar Patnana, Subbaiah Chary Nimmagadda

**Affiliations:** Department of Medicine A, Hematology, Oncology and Pneumology, University Hospital Muenster, Muenster, Germany

**Keywords:** Target identification, Molecular medicine

Recent advances demonstrated numerous molecular mechanisms of metformin based on the dosage and duration, yet our understanding is limited. Using a photoactive metformin probe Ma et al.^1^ identified presenilin enhancer 2 (PEN2) as its interaction partner and demonstrated its contribution to AMP-activated protein kinase (AMPK) activation.^1^

Metformin is a widely used anti-diabetic for managing blood glucose levels in patients with type 2 diabetes mellitus (T2D). Owing to its pleiotropic modes of action, its role in other pathophysiological conditions including metabolic complications, cancer, cardiovascular diseases, inflammation, ageing, gut microbiota, neuroprotection and COVID-19 were reported. It is widely accepted that metformin inhibits mitochondrial complex I of the electron transport chain and results in a decrease in ATP synthesis stimulating AMPK, inhibiting hepatic gluconeogenesis, increasing insulin sensitivity in the liver and intensifying glucose absorption in the muscles. In addition, AMPK-independent role for metformin in cell proliferation, glycolysis and oxygen consumption rate, tumour suppression and blocking of important pathways as mTOR were reported.

**Fig. 1:**
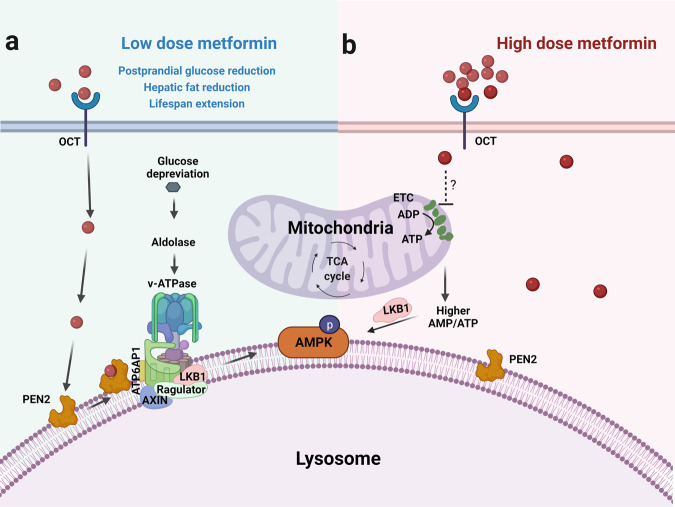
Schematic illustration of low- and high-dose metformin-induced signalling events leading to AMPK activation. **a** (green): Low-dose metformin-PEN2 and low-glucose sensor, aldolase signalling intersect at v-ATPase and results in AMPK activation. This is associated with postprandial glucose reduction, hepatic fat reduction, and life span extension. **b** (red): High-dose metformin activates AMPK signalling independent of the PEN2-ATP6AP1 axis and triggers a spectrum of other pathways. AMP adenosine monophosphate, ATP adenosine triphosphate, AMPK AMP-activated protein kinase, ATP6AP1 accessory factor of v-ATPase, ETC electron transport chain, LKB1 liver kinase B1, OCT organic cation transporters, PEN2 presenilin enhancer 2. The figure was created with a paid license on BioRender.com

Zhang and colleagues previously found that metformin activated AMPK by inducing lysosomal translocation of AXIN and LKB1 to form the Ragulator-v-ATPase-AXIN/LKB1-AMPK super-complex (Fig. 1).^2^ To better understand this and to evaluate how low-dose (5–30 μM) metformin activated AMPK, Ma et al.^1^ generated photoactive metformin probes and exploited an affinity-based approach to identify its potential targets in lysosomal protein extracts. Using mouse embryonic fibroblasts (MEFs) and shRNA silencing strategy, PEN2 was identified as a binding partner of metformin (from a potential pool of 113 proteins). Depletion of PEN2 did not affect the transport of metformin but blocked low-dose metformin-induced AMPK activation and vacuolar ATPase (v-ATPase) inhibition. Imaging techniques (confocal, stochastic optical reconstruction, and APEX tag-based transmission electron microscopy) further hinted at a distinct pool of lysosomal localised PEN2 for mediating metformin-induced AMPK activation. Mass spectrometry and in silico studies identified PEN2 resident tyrosine-47, phenylalanine-35 and glutamate-40 as critical residues for metformin interaction. Furthermore, to understand how metformin directs PEN2 to interact with and inhibited v-ATPase, the authors analysed PEN2 immunoprecipitates via mass spectrometry. Of the 123 proteins, ATP6AP1 (accessory factor of v-ATPase) received consideration since PEN2-ATP6AP1 interaction could be authenticated. Characterisation of the ATP6AP1 domains determined that its transmembrane domain (AA 420–440) was critical PEN2 binding. The author’s group earlier reported that under glucose deprivation, AXIN, Ragulator and v-ATPase were essential for lysosomal AMPK activation. However, in PEN2 and ATP6AP1 depleted MEFs, lysosomal translocation and formation of AXIN based complex were blocked. The observations thus position PEN2-ATP6AP1 upstream of AXIN and Ragulator in priming the signalling events. Of note, high-dose metformin (>100 µM) bypassed the requirement of PEN2, AXIN and LAMTOR1 for AMPK activation. These findings thus shed light on how metformin-PEN2 were recruited to ATP6AP1 to relay low-dose metformin-driven v-ATPase inhibition/AMPK activation (Fig. 1).

On a functional level, the authors evaluated the physiological role of PEN2-ATP6AP1 interaction in mediating the beneficial effects of metformin in animal models. While PEN2 depletion in the mice intestine demonstrated impaired postprandial glucose-lowering effects of metformin, hepatic-specific depletion of PEN2 led to significantly impaired AMPK activation similar to those observed in *Ampka-*knockout (ko) mice. Additionally, metformin-induced reduction of hepatic triglycerides and glucose intolerance in obese mice were blocked. Re-expression of the truncated variant of ATP6AP1^Δ420–440^ in livers of *Atp6ap1*-ko mice failed to rescue these effects. Finally, the authors investigated whether the metformin-induced longevity effect depends on PEN2 and ATP6AP1. In line with the literature, metformin improved longevity and retarded ageing via AMPK activation in mice and *Caenorhabditis elegans* (*C. elegans*) models. Strikingly, in either model’s depletion of PEN2, ATP6AP1 or re-expression of ATP6AP1^Δ420–440^ failed to rescue metformin-induced lysosomal AMPK activation and longevity effect. The observations thus emphasised the important contributions of PEN2-ATP6AP1 in mediating the effect of metformin on decreasing glucose levels, hepatic fat content and improving longevity.

Metformin’s response in T2D patients greatly varies and 20–30% of patients develop gastrointestinal related side effects leading to its discontinuation. Besides, genetic polymorphisms and downregulation of the expression of organic cation transporters (OCT1-3) in malignancies such as kidney and liver cancers markedly hinder the uptake of metformin. On the other hand, metformin’s anti-neoplastic role is beginning to be appreciated. Recent reports including ours demonstrated metformin together with chemotherapeutic agents induced a pronounced cytotoxic effect.^3^ Metformin although it reduced cancer risk and improved prognosis in several cancers, the outcomes were relatively heterogeneous since high-dose metformin is challenging to achieve without severe side effects in patients.^4^ Thus therapeutic exploitation of the PEN2-ATP6AP1 axis together with metformin alternatives and neoadjuvant chemotherapies respectively might promise an improved outcome in T2D and cancer patients. In accordance with Ma et al. preclinical studies in animal models and human clinical trials revealed the favourable effect of metformin on longevity and anti-ageing. These effects were again heterogeneous and often associated with high-dose metformin. Preliminary results from studies such as Metformin in Longevity Study (MILES) indicated that metformin conferred anti-ageing benefits via modulating transcriptional changes, but these observations remained controversial in subjects free of disease.^5^ It will therefore be interesting to examine if PEN2-ATP6AP1 signalling contributed to metformin’s role in anti-ageing and longevity.

Taken together, the work by Ma et al. elegantly identifies the PEN2-ATP6AP1-AMPK axis and sheds light on a novel molecular mechanism and associated benefits of low-dose metformin in lysosomes in an AMP-independent manner. The findings represent a breakthrough in the exploration of how different doses of metformin resulted in AMPK activation and lays the groundwork for future research into novel therapeutics in T2D, ageing and cancer treatment. Despite these encouraging results, with T2D incidence increasing in older people, it is important to see if the observations could be validated in older and appropriate diabetic mice models (ideally in a chronic setting). Besides, recapitulation of human T2D complications and biodistribution of metformin and its transporters is always challenging. Ma et al.^1^ also showed that high-dose metformin activated a large pool of cellular AMPK independent of the lysosomal PEN2–v-ATPase-AMPK pathway. Furthermore, our understanding of metformin-induced AMPK-dependent or -independent signalling is limited and deserves attention for future research to avoid high-dose metformin-induced substantial adverse effects.
